# Energy Efficient SWIPT Based Mobile Edge Computing Framework for WSN-Assisted IoT

**DOI:** 10.3390/s21144798

**Published:** 2021-07-14

**Authors:** Fangni Chen, Anding Wang, Yu Zhang, Zhengwei Ni, Jingyu Hua

**Affiliations:** 1 College of Information Engineering, Zhejiang University of Technology, Hangzhou 310012, China; chenfangni@zust.edu.cn (F.C.); yzhang@zjut.edu.cn (Y.Z.); 2 School of Information and Electronic Engineering, Zhejiang University of Science and Technology, Hangzhou 310012, China; 3 School of Information and Electronic Engineering, Zhejiang Gongshang University, Hangzhou 310018, China; anding_704@zjgsu.edu.cn (A.W.); zhengwei.ni@zjgsu.edu.cn (Z.N.)

**Keywords:** mobile edge computaing, simultaneous wireless information and power transfer, energy minimization, 5G, wireless sensing network, IoT

## Abstract

With the increasing deployment of IoT devices and applications, a large number of devices that can sense and monitor the environment in IoT network are needed. This trend also brings great challenges, such as data explosion and energy insufficiency. This paper proposes a system that integrates mobile edge computing (MEC) technology and simultaneous wireless information and power transfer (SWIPT) technology to improve the service supply capability of WSN-assisted IoT applications. A novel optimization problem is formulated to minimize the total system energy consumption under the constraints of data transmission rate and transmitting power requirements by jointly considering power allocation, CPU frequency, offloading weight factor and energy harvest weight factor. Since the problem is non-convex, we propose a novel alternate group iteration optimization (AGIO) algorithm, which decomposes the original problem into three subproblems, and alternately optimizes each subproblem using the group interior point iterative algorithm. Numerical simulations validate that the energy consumption of our proposed design is much lower than the two benchmark algorithms. The relationship between system variables and energy consumption of the system is also discussed.

## 1. Introduction

### 1.1. Backgroud

The 5G enabled Internet of Things (5G-IoT) [1,2,3,4] connects the real world with the internet world and human civilization is currently transforming from informatization to intelligence. The four abilities of 5G communication system, namely massive capacity, ultra-low latency, high reliability and extensive connection, are the key driving forces for the development of IoT [5,6]. The integration of 5G and WSN-assisted IoT not only strengthens the connection between the real world and the internet world, but also widens the scope of IoT services such that IoT can not only serve the smart city [7] but also penetrate into agriculture [8], medical care [9], transportation [10], industry [11] and other fields [12]. However, WSN-assisted IoT is facing grand challenges and the huge amount data traffic brought by great number of IoT devices and sensors can impose an enormous burden on the network, resulting in higher service delays and reduced quality of service (QoS) [13].

Although the current terminal devices are equipped with high-performance hardware, it is still difficult to meet the needs of computing intensive tasks, especially in the case of ensuring low power consumption and low latency. Mobile Edge Computing (MEC) technology is considered as a crucial solution for 5G-IoT [14,15]. With the help of MEC, terminal devices can upload part of or all of the computing tasks to the edge computing platform for computing so as to reduce their own computing pressure and energy consumption, improve the computing efficiency and performance and bring better QoS [16,17,18]. The authors of [19] studied how the MEC enabled industrial verticals in 5G. Yang et al. [20] analyzed the main features of MEC in the context of 5G and IoT and presented several fundamental key technologies that enable MEC to be applied in 5G and IoT.

Despite the fact that MEC brings great benefits for IoT, it still faces an important problem: how can we effectively and conveniently extend the lifetime of IoT devices in the network? Simultaneous wireless information and power transfer (SWIPT) is considered as the key technology to solve this problem. The principle of SWIPT system is that RF signals can carry energy and information at the same time. It was first proposed by the author of [21] in 2008. Rui Zhang et.al. [22] proposed two practical SWIPT receivers, which are the time switching (TS) receiver and power splitting (PS) receiver. The TS receiver divides the time slot into two parts, the RF signal received in the first part of time is used for information demodulation and then the signal collected in the remaining time is used for energy harvesting. The PS receiver divides the received RF signal into two parts and then transmits them to the energy collector and information demodulator, respectively, so that the information demodulation and energy harvesting can be realized simultaneously. Since then, a lot of researches focus on the design and performance of the SWIPT systems [23,24]. The author of [25] proposes a more complex dynamic energy splitting receiver based on the above two receivers framework. The author of [26] studied the trade off between the harvested energy and forward data in SWIPT sensor networks. Tang et al. [27] proposed a TS receiver design to maximize the energy efficiency in MIMO channels for IoT.

### 1.2. Related Works

As both SWIPT and MEC technologies benefit the IoT system, MEC deployed WSN IoT network design with SWIPT has attracted increasing attention. In such new framework, the communication and computation resource allocation as well as wireless energy harvesting scheme are crucial for maximizing the system performance. The authors of [8] studied an energy efficiency optimization scheme for OFDM transmission WSN in smart agriculture. By jointly optimizing the power allocation and the pairing of subcarriers, the optimization scheme can help to solve the problem of energy deficiency. An achievable rate maximization problem was discussed in [28] for multiuser satellite IoT system with SWIPT and MEC to overcome the limitation in battery capacity and computing capability of IoT terminals. In [29], a UAV-enabled wireless powered MEC system was investigated, where the offloading modes were optimized to reach the maximum computation rate under the power constraint and the UAV speed constraint. The authors of [30] extended multi-access edge computing to support the long range (LoRa) system for IoT applications. The novel framework allowed dynamic IoT deployment at the edge and life cycle management.

With the development of artificial intelligence technology, reinforcement learning (RL) methods are used to solve various communication problems in 5G and IoT systems. Aiming at minimizing the difference between the distributed and demanded throughput for each user, ref. [31] presented a novel deep reinforcement learning (DRL) scheme, which satisfied the user requirements by power regulation. In [32], a RL based offloading scheme was studied to select the edge device and the offloading rate for IoT devices. The distinguished merit of this scheme is that the offloading policy can be optimized without knowledge required in traditional schemes. A hybrid-decision-based DRL approach is proposed in [33] to provide coordinated decisions of dynamic offloading scheme for multi-device multi-server MEC-IoT systems with energy harvesting devices.

### 1.3. Contributions

In this paper, we investigate energy consumption minimization for SWIPT based mobile edge computing in WSN assisted IoT System by using the optimization process. It is a continuation of our previous work [34] that focused on the cellular system. However, this study focus on the WSN assisted IoT network. Different from existing works, an in-depth research is carried to analyze the effects of computation task size, mobile node (including wireless sensor node) number, antenna number and energy harvest weight factor on the system energy consumption. The novelties of this work are summarized as follows.

(1) We design a novel WSN assisted IoT System, which integrates a MEC-deployed and FD-deployed anchor node (AN) and multiple SWIPT-equipped mobile nodes (MNs). The research problem of modeling is completely different from our previous work since the transmission conditions and requirements between cellular communication system and wireless sensor network are different. We aim to achieve the minimum energy consumption by optimizing CPU frequency, power allocation, offloading weight factor and SWIPT weight factor. Moreover, given the analysis of uplink, we provide the closed form expression of downlink rate and obtain the downlink transmission delay. Moreover, we optimize the SWIPT weight factor, which will affect the energy consumption of downlink harvesting and then affect the total system energy consumption. A more reasonable expression of harvesting energy is provided, which is based on the SWIPT weight factor and downlink time delay. In other words, the uplink and downlink parameters are jointly optimized.

(2) We formulate a more practical WSN energy minimization problem by jointly optimizing the key decision variables in the system. Since the multiple variables to be optimized are coupled and the original problem is non-convex, the optimization is quite challenging. An efficient algorithm called alternate group iteration optimization (AGIO) is proposed. We decompose the decision variables into three groups and divide the original problem into three subproblems. Then, we alternately optimize each subproblem using the interior point iteration method until the convergence.

The rest of the paper is organized as follows. Section 2 describes the system model and analyzes the transmission process. Section 3 formulates the energy minimization problem. Section 4 and Section 5 present the algorithms to solve the problem and provides simulation results. Section 6 concludes the paper.

## 2. System Model

**Figure 1:**
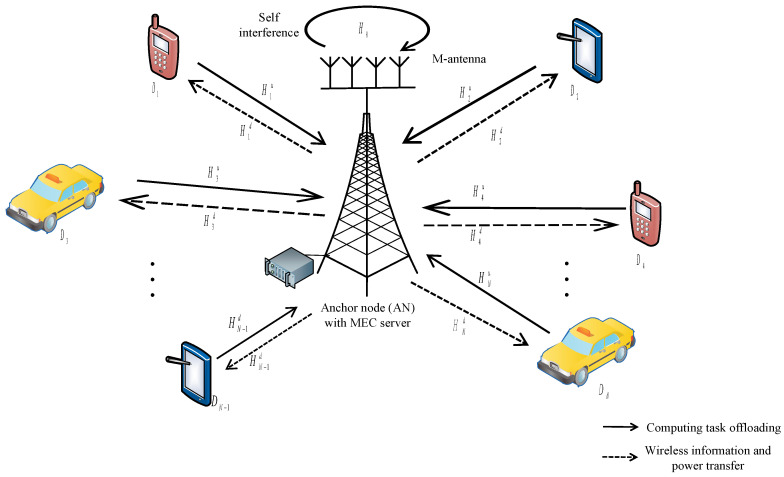
SWIPT-MEC enabled WSN assisted IoT System Model.

Let us consider a SWIPT-MEC enabled WSN assisted IoT system as demonstrated in Figure 1. There are *N* mobile nodes (MNs) including wireless sensor nodes denoted as $\left\{D_{1},D_{2},\cdots ,D_{N}\right\}$ that are overwhelmed with computation tasks and one *M*-antenna Full Duplex enabled anchor node (AN). Each MN deploys single antenna and a power splitting (PS) SWIPT equipment to harvest energy. The PS receiver is capable of switching between energy harvesting (EH) state and information decoding (ID) state. The anchor node is equipped with a MEC server that can help MNs with the enormous amount of computation tasks.

Assume that each MN can divide its computation task into two parts and one is for local computing and the other is for offloading to the MEC enabled AN. The total computation task size of $D_{i}$ is represented as $L_{i}$ bits and the offloading computation task size is $L_{i}^{u}$ bits, which satisfies $L_{i}^{u}=\alpha_{i}L_{i}$, where $0\leq \alpha_{i}\leq 1$ is a offloading weight factor. Since MN can decide how much computation task will be offloaded to AN, $\alpha_{i}$ is a variable to be optimized to achieve better performance.

The operation processes of the system can be illustrated in the following steps.

(1) During the uplink process, MN $D_{i}$ (i∈{1,⋯,N} ) offloads the computation task $L_{i}^{u}$ to the MEC server at AN.

(2) When the MEC server receives the offloading task, it immediately implements the computation task. Due to the strong computation ability, MEC server can finish the offloading computation task in a short time, which can be ignored compared to the other operation times.

(3) Since AN deploys FD technology when MN $D_{i}$ is offloading, AN can simultaneously download the computation result $L_{j}^{d}$ from the MEC server to MN $D_{j}$ (j∈{1,⋯,N}, j≠i ), which shares the same frequency as the uplink MN $D_{i}$. The computation result satisfies $L_{j}^{d}=\beta_{j}L_{i}^{u}$, where $0\leq \beta_{j}\leq 1$ is a weight factor.

(4) After $D_{j}$ receives the computation result, the PS receiver will perform energy harvesting and information decoding according to the received RF signal.

(5) Mobile node $D_{i}$ will perform local computing on the remaining computaion task when it finishes the offloading.

The above processes can be divided into three phases: the offloading phase, downloading phase and local computing phase. They are described in detail in the following analysis.

### 2.1. Offloading Phase

In the offloading phase, MNs $\left\{D_{1},D_{2},\cdots ,D_{N}\right\}$ that are overwhelmed by the computation tasks will offload part of the tasks to AN. Without loss of generality, AN receives the offloading computation task $L_{i}^{u}$ from MN $D_{i}$ during the time interval $t_{i}^{u}$. Meanwhile, AN simultaneously downloads computation result $L_{j}^{d}$ to $D_{j}$, where i∈{1,⋯,N},j∈{1,⋯,N},i≠j. Thus, the received signal at AN is the following:

(1) $$\begin{array}{c} y_{i}^{u}=\sqrt{p_{i}^{u}}H_{i}^{u}s_{i}^{u}+\sqrt{\eta_{j}}H_{0}\left(\sqrt{p_{j}^{d}}s_{j}^{d}\right)+n_{\mathrm{AN}}, \end{array}$$

where $p_{i}^{u}$ and $s_{i}^{u}$ are the transmitted power and transmitted signal of $D_{i}$, $p_{j}^{d}$ and $s_{j}^{d}$ are the transmitted power and the computation result signal transmitted from AN to MN $D_{j}$. The two transmitted signals are assumed with normalized power, i.e., ${|s_{i}^{u}|}^{2}=1$ and $|s_{j}^{d}{|}^{2}=1$. $H_{i}^{u}\in C^{M\times 1}$ is the uplink channel from $D_{i}$ to AN and $H_{0}\in C^{M\times M}$ is the self-interference channel induced by FD transmission. $\eta_{j}$ is the residual self-interference (RSI) coefficient. The received noise is $n_{\mathrm{AN}}∼CN\left(0,\sigma_{AN}^{2}I_{M}\right)$.

According to the above expression, only the first part on the right side of Equation (1) contains the target offloading task $s_{i}^{u}$, the seond part is the RSI due to FD transmission of AN and the third part is the additive white Gaussian noise (AWGN) at AN. Thus, the signal to interference plus noise ratio (SINR) can be represented as the following:

(2) $$\begin{array}{c} \gamma_{i}^{u}=\frac{p_{i}^{u}tr\left\{H_{i}^{u}{\left(H_{i}^{u}\right)}^{H}\right\}}{\eta p_{j}^{d}tr\left\{H_{0}H_{0}^{H}\right\}+\sigma_{AN}^{2}}, \end{array}$$

where tr{.} represents the matrix trace. Then, we can obtain the transmission rate as the following:

(3) $$\begin{array}{c} R_{i}^{u}=Blog_{2}\left(1+\gamma_{i}^{u}\right), \end{array}$$

where *B* denotes the bandwidth allocated. Let $R_{min}^{u}$ represent the minimum uplink transmission rate requirement. Therefore, we can obtain the first constraint condition in this system model shown as the following:

(4) $$\begin{array}{c} R_{i}^{u}\geq R_{min}^{u}. \end{array}$$

Meanwhile, we can calculate the transmission time for offloading as follows.

(5) $$\begin{array}{c} t_{i}^{u}=\frac{L_{i}^{u}}{R_{i}^{u}}. \end{array}$$

Thus, the energy consumed by $D_{i}$ at the offloading phase can be expressed as the following:

(6) $$\begin{array}{c} E_{i}^{off}=p_{i}^{u}\frac{L_{i}^{u}}{R_{i}^{u}}, \end{array}$$

and the resulting transmission energy consumed by all MNs is provided by the following.

(7) $$\begin{array}{c} E^{off}=\sum_{i=1}^{N}E_{i}^{off}. \end{array}$$

### 2.2. Downloading Phase

Since AN is equipped with FD technology, it can download the computation result to MN $D_{j}$ and receive offloading task from other MN $D_{i}$ simultaneously. Thus, the received signal at $D_{j}$ is described as the following:

(8) $$\begin{array}{c} y_{j}^{d}=\sqrt{p_{j}^{d}}H_{j}^{d}s_{j}^{d}+n_{j}^{d}, \end{array}$$

where $p_{j}^{d}$ is the transmitted power AN uses for downloading the computation result to $D_{j}$. $n_{j}^{d}$ is the AWGN with power $\sigma_{j}^{2}$.

Here, we suppose that the co-channel interference can be canceled perfectly in the receiver for the sake of simplicity.

Then, the signal to interference plus noise ratio (SINR) and the transmission rate at MN $D_{j}$ are the following.

(9) $$\begin{array}{c} \gamma_{j}^{d}=\frac{p_{j}^{d}tr\left\{H_{j}^{d}{\left(H_{j}^{d}\right)}^{H}\right\}}{\sigma_{j}^{2}}, \end{array}$$

(10) $$\begin{array}{c} R_{j}^{d}=Blog_{2}\left(1+\gamma_{j}^{d}\right). \end{array}$$

Let $R_{min}^{d}$ represent the minimum downlink transmission rate requirement and we can obtain another constraint condition described as follows.

(11) $$\begin{array}{c} R_{j}^{d}\geq R_{min}^{d}. \end{array}$$

Meanwhile, we can calculate the latency of the downlink transmission as follows.

(12) $$\begin{array}{c} t_{j}^{d}=\frac{L_{j}^{d}}{R_{j}^{d}}. \end{array}$$

The PS receiver at $D_{j}$ then divides the received RF signal into two parts; the $\theta_{j}$ (0≤θ≤1) part is used for energy harvesting, while the rest $\left(1-\theta_{j}\right)$ part is used for information decoding. We can obtain the harvest energy of $D_{j}$ as follows.

(13) $$\begin{array}{c} E_{j}^{hav}=\theta_{j}\left(p_{j}^{d}tr\left\{H_{j}^{d}{\left(H_{j}^{d}\right)}^{H}\right\}+\sigma_{j}^{2}\right)t_{j}^{d}. \end{array}$$

Meanwhile, the energy consumption of AN can be calculated as the following.

(14) $$\begin{array}{c} E^{AN}=\sum_{j=1}^{N}p_{j}^{d}t_{j}^{d}. \end{array}$$

### 2.3. Local Computation Phase

After offloading, MN operates on the remaining computation task. Let $f_{i}^{n}$ denote the CPU frequency needed for the n-th CPU cycle of $D_{i}$. The following constraint condition should be met:

(15) $$\begin{array}{c} 0\leq f_{i}^{n}\leq f_{i}^{max},\forall i \end{array}$$

where $f_{i}^{max}$ is the maximum CPU frequency of $D_{i}$. Then, the time for local computation of $D_{i}$ is the following:

(16) $$\begin{array}{c} t_{i}^{local}=\sum_{n=1}^{C(L_{i}-L_{i}^{u})}\frac{1}{f_{i}^{n}}, \end{array}$$

where *C* is the CPU cycles required for computing 1-bit of data. The energy consumption of local computation is given by the following:

(17) $$\begin{array}{c} E_{i}^{loc}=\sum_{n=1}^{C(L_{i}-L_{i}^{u})}\kappa {\left(f_{i}^{n}\right)}^{2}, \end{array}$$

where κ is the effective capacitance coefficient based on the chip architecture [35]. Thus, we can obtain the total local energy consumption as follows.

(18) $$\begin{array}{c} E^{loc}=\sum_{i=1}^{N}E_{i}^{loc}. \end{array}$$

## 3. Problem Formulation

After we analyze the transmission process of the system, we can formulate an problem which can optimize the system performance. This paper aims at minimizing the total energy consumption of the system, while simultaneously ensuring the transmission requirements. The total energy consumption of the system contains AN energy consumption $E^{AN}$, the offloading energy consumption $E^{off}$ and the energy consumption of local computation $E^{loc}$. In addition, we need to remove the harvest energy $E^{hav}$ MNs can obtain. Based on Equations (7), (13), (14) and (18), we can write the total energy consumption of the system as the following.

(19) $$\begin{array}{cc} E^{total} & =E^{AN}+E^{off}+E^{loc}-E^{uh}\\ \; & =\sum_{j=1}^{N}p_{j}^{d}\frac{L_{j}^{d}}{R_{j}^{d}}+\sum_{i=1}^{N}p_{i}^{u}\frac{L_{i}^{u}}{R_{i}^{u}}+\sum_{i=1}^{N}\sum_{n=1}^{C(L_{i}-L_{i}^{u})}\kappa {\left(f_{i}^{n}\right)}^{2}\\ \; & -\sum_{j=1}^{N}\theta_{j}\left(p_{j}^{d}tr\left\{H_{j}^{d}{\left(H_{j}^{d}\right)}^{H}\right\}+\sigma_{j}^{2}\right)\frac{L_{j}^{d}}{R_{j}^{d}} \end{array}$$

Finally, the problem can be described as follows:

(20) $$\begin{array}{cc} \left(P1\right) & \min_{f,p^{u},p^{d},\alpha ,\theta }E^{total}\\ \; & s.t.\left\{\begin{array}{c} C1:0\leq \alpha ,\theta \leq 1\\ C2:R_{i}^{u}\geq R_{min}^{u},\forall i\\ C3:R_{j}^{d}\geq R_{min}^{d},\forall j\\ C4:p_{i}^{u}\leq P_{max}^{u},\forall i\\ C5:p_{j}^{d}\leq P_{max}^{d},\forall j\\ C6:0\leq t_{i}^{local}\leq t_{i}^{u},\forall i\\ C7:0\leq f_{i}^{n}\leq f_{i}^{max},\forall i \end{array}\right. \end{array}$$

where f$=[f_{1},f_{2},\cdots ,f_{N}]$, $p^{u}=\left[p_{1}^{u},p_{2}^{u},\cdots ,p_{N}^{u}\right]$, $p^{d}=\left[p_{1}^{d},p_{2}^{d},\cdots ,p_{N}^{d}\right]$, $\alpha =[\alpha_{1},\alpha_{2},\cdots ,\alpha_{N}]$ and $\theta =[\theta_{1},\theta_{2},\cdots ,\theta_{N}]$.

In problem P1, *C*1 provides the constraints on weight factors. *C*2 and *C*3 imply that the offloading rate and downloading rate should not be less than the QoS requirements $R_{min}^{u}$ and $R_{min}^{d}$, respectively. *C*4 and *C*5 indicate the uplink and downlink transmission power limits $P_{max}^{u}$ and $P_{max}^{d}$. *C*6 denotes that the time of the local computation should be no more than the time of the offloading phase, otherwise it is better to offload all the tasks. *C*7 indicates the CPU frequency constraint according to Equation (15).

## 4. The Proposed Algorithm

In this section, we will solve the formulated problem in steps. The problem P1 is full of challenges since both the objective function and the constraints are non-convex. Although the variables that need to be optimized are all coupled in P1, we find that the CPU frequency f is the least relevant variable compared to other variables. We divide P1 into the following three subproblems. (i) Local computation optimization is as follows: In this subproblem, we obtain the optimal CPU frequency using the scheme in [36]. (ii) Power optimization is as follows: When f and the weight factors α, θ are fixed, we can use the interior point algorithm to solve the problem. (iii) Weight factor optimization is as follows: After frequency and power optimization are completed, interior point algorithm can be used again to obtain the optimal weight factors. The three subproblems should be optimized alternately by the iteration method.

### 4.1. Local Computation Optimization

Inspired by [36], the optimal CPU frequency should satisfy the following.

(21) $$\begin{array}{c} f_{i}^{1}=f_{i}^{2}=\cdots =f_{i}^{C(L_{i}-L_{i}^{u})}=\bar{f_{i}}. \end{array}$$

The above equation reveals the CPU frequency should maintain the same in each cycle as $\bar{f_{i}}$. Suppose the other four variables have been optimized, the initial problem (P1) can be reformulated as follows:

(22) $$\begin{array}{cc} \left(P2\right) & \min_{\bar{f}}E+\sum_{i=1}^{N}C(L_{i}-L_{i}^{u})\kappa {\left(\bar{f_{i}}\right)}^{2}\\ \; & s.t.\left\{\begin{array}{c} C6:0\leq \frac{C(L_{i}-L_{i}^{u})}{\bar{f_{i}}}\leq t_{i}^{u},\forall i\\ C7:0\leq \bar{f_{i}}\leq f_{i}^{max},\forall i \end{array}\right. \end{array}$$

where $E=E^{AN}+E^{off}-E^{hav}$, $\bar{f}=\left[\bar{f_{1}},\bar{f_{2}},\cdots ,\bar{f_{N}}\right]$. According to the optimization objective function, the energy consumption increases monotonically with $\bar{f_{i}}$. In other words, $\bar{f_{i}}$ has to be the smallest value to achieve the minimum energy consumption. Thus, from the constraint C6, we can obtain the following.

(23) $$\begin{array}{c} f_{i}^{opt}=\frac{C(L_{i}-L_{i}^{u})}{t_{i}^{u}}. \end{array}$$

Then, by replacing $f_{i}^{n}$ with $f_{i}^{opt}$ in Equation (16), we can obtain the local energy consumption of user $D_{i}^{u}$ as follows.

(24) $$\begin{array}{c} E_{i}^{loc}=\frac{\kappa C^{3}{\left(L_{i}-L_{i}^{u}\right)}^{3}}{{\left(t_{i}^{u}\right)}^{2}}. \end{array}$$

According to Equation (16), we find that the effect of CPU frequency on energy consumption can be transformed to the effect of offloading ratio and transmitting power. Therefore, we only need to focus on the optimization of these two parameters in the following steps.

### 4.2. Power Optimization

After the CPU frequency has been optimized and with the assumption that the two weight factors have been optimized, problem P1 now can be rewritten as the following.

(25) $$\begin{array}{cc} \left(P3\right) & \min_{p^{u},p^{d}}\sum_{j=1}^{N}p_{j}^{d}\frac{L_{j}^{d}}{R_{j}^{d}}+\sum_{i=1}^{N}p_{i}^{u}\frac{L_{i}^{u}}{R_{i}^{u}}+\sum_{i=1}^{N}\frac{\kappa C^{3}{\left(L_{i}-L_{i}^{u}\right)}^{3}}{{\left(t_{i}^{u}\right)}^{2}}\\ \; & \;\;\;\;-\sum_{j=1}^{N}\theta_{j}\left(p_{j}^{d}tr\left\{H_{j}^{d}{\left(H_{j}^{d}\right)}^{H}\right\}+\sigma_{j}^{2}\right)\frac{L_{j}^{d}}{R_{j}^{d}}\\ \; & s.t.\begin{array}{c} C2,C3,C4,C5. \end{array} \end{array}$$

For problem P3, the second-order derivative of each variable, i.e., $p_{1}^{u},p_{2}^{u},\cdots ,p_{N}^{u}$ and $p_{1}^{d},p_{2}^{d},\cdots ,p_{N}^{d}$ of the objective function is zero. Moreover, all constraints are linear. Thus, the standard interior point algorithm can be applied to this convex problem and the optimal solution can be achieved.

### 4.3. Weight Factor Optimization

With the optimized variables $f,p^{u},p^{d}$, the problem can can be expressed as the following:

(26) $$\begin{array}{cc} \left(P4\right) & \min_{\alpha ,\theta }\sum_{j=1}^{N}p_{j}^{d}\frac{\beta_{j}\alpha_{i}L_{i}}{R_{j}^{d}}+\sum_{i=1}^{N}p_{i}^{u}\frac{\alpha_{i}L_{i}}{R_{i}^{u}}+\sum_{i=1}^{N}\frac{\kappa C^{3}{\left(1-\alpha_{i}\right)}^{3}{\left(L_{i}\right)}^{3}}{{\left(t_{i}^{u}\right)}^{2}}\\ \; & \;\;\;\;-\sum_{j=1}^{N}\theta_{j}\left(P_{j}^{d}+\sigma_{j}^{2}\right)\frac{\beta_{j}\alpha_{i}L_{i}}{R_{j}^{d}}\\ \; & s.t.\begin{array}{c} C1:0\leq \alpha ,\theta \leq 1 \end{array} \end{array}$$

where $P_{j}^{d}=p_{j}^{d}tr\left\{H_{j}^{d}{\left(H_{j}^{d}\right)}^{H}\right\}$. Similar to problem P3, problem P4 is a convex problem which can be solved by the interior point algorithm.

Based on the above discussion, the proposed AGIO algorithm can effectively solve the optimization problem P1, which is illustrated in Algorithm 1.
**Algorithm 1** Alternate Group Iterative Optimization**Input:**$N,M,B,H^{u},H^{d},H_{0},C,\kappa ,L,\sigma_{j}^{2},\sigma_{AN}^{2}$, $\beta ,\eta ,P_{max}^{u},P_{max}^{d},R_{min}^{u},R_{min}^{d}$**Output:** optimal solutions $f^{*},p^{(u)*},p^{(d)*},\alpha^{*},,\theta^{*}$  1: Set iteration number n=1.  2: Set maximum iteration number $I_{max}$.  3: Set the initial values: $p^{u(0)},p^{d(0)},\alpha^{\left(0\right)},\theta^{\left(0\right)}$.  4: **While**
$n\leq I_{max}$  5:        Solve problem P2 to obtain the optimal f;  6:        Solve problem P3 to obtain the optimal $\left(p^{u(n)},p^{d(n)}\right)$;  7:        Solve problem P4 to obtain the optimal $\left(\alpha^{\left(n\right)},\theta^{\left(n\right)}\right)$  8:        Set n=n+1;  9: **End While**.

## 5. Simulation and Analysis

### 5.1. Simulation Results

In this section, the research work above is simulated and the effects of different variables on the system performance are investigated. The simulation parameters are summarized as follows: the bandwidth is 5 MHz; the upper limits of transmitting power are $p_{1,max}^{u}=p_{2,max}^{u}=\cdots =p_{N,max}^{u}=5$ W and $p_{1,max}^{d}=p_{2,max}^{d}=\cdots =p_{N,max}^{d}=20$ W; the weight factors are $\beta_{1}=\beta_{2}=\cdots =\beta_{N}=1$; and the noise power is $\sigma_{AN}^{2}=\sigma_{j}^{2}=-120$ dBm, ∀j. We also set the chip effective capacitance coefficient to $\kappa =10^{-20}$ and the CPU cycles are $C=10^{3}$ cycles/bit. We choose a random Rayleigh fading channel model for all the channel matrix in the simulation. Moreover, we apply two benchmark algorithms to compare with the proposed AGIO algorithm.

(1) The fixed-variable (FV) algorithm: The variables $p^{u},p^{d},\alpha ,\theta$ are fixed at initial values.

(2) The full-offloading (FO) algorithm: MNs upload all computation tasks to the MEC server. Thus, the local computation task is zero, the CPU frequency of local computation is $f_{1}=f_{2}=\cdots =f_{N}=0$ and the offloading weight factor is $\alpha_{1}=\alpha_{2}=\cdots =\alpha_{N}=1$.

**Figure 2:**
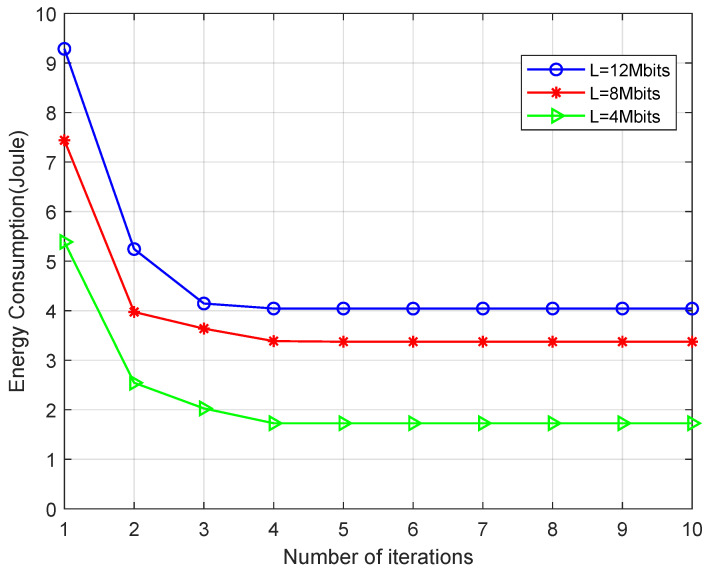
Convergence behavior of the proposed AGIO algorithm.

Figure 2 demonstrates that convergence performance of the proposed AGIO algorithm under different computation task size L (4 Mbits, 8 Mbits and 12 Mbits ) with 6 MNs and the antenna number of AN is 6. As shown in the figure, the algorithm converges after five iterations under varying L. This proves the effectiveness of our algorithm.The convergence curves also indicate that the energy consumption increases with computation task size L.

**Figure 3:**
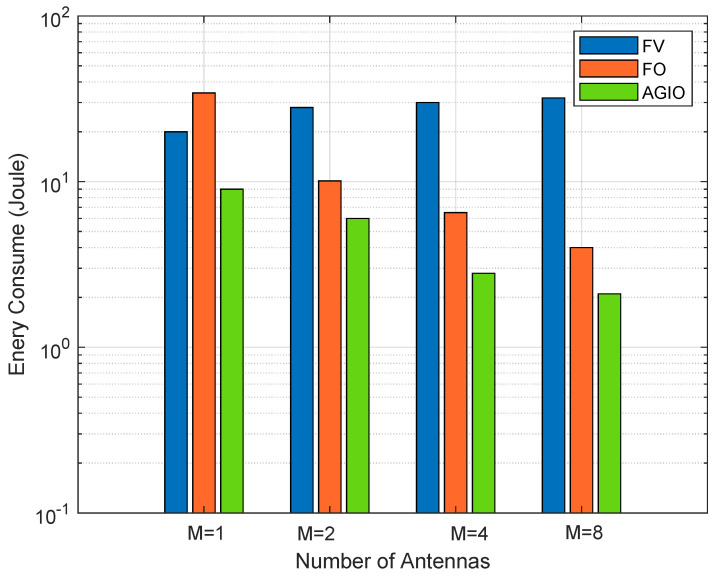
Comparison of energy consumption with a different number of antennas.

Figure 3 compares the system energy consumption of the three different algorithms under a different number of AN antennas *M*. The proposed AGIO has the minimum system energy consumption in all the scenarios, which shows the performance superiority of the AGIO algorithm. The number of antennas of the FV algorithm has the least impact on energy consumption. It increases gently with the number of AN antennas. On the other hand, for FO algorithm and the proposed AGIO algorithm, the energy consumption is greatly reduced with *M*. As a result, our algorithm indicates greater performance improvement in energy consumption when the number of the AN antennas increases. Thus, we can conclude that the proposed AGIO algorithm is suitable for multi-antenna AN.

**Figure 4:**
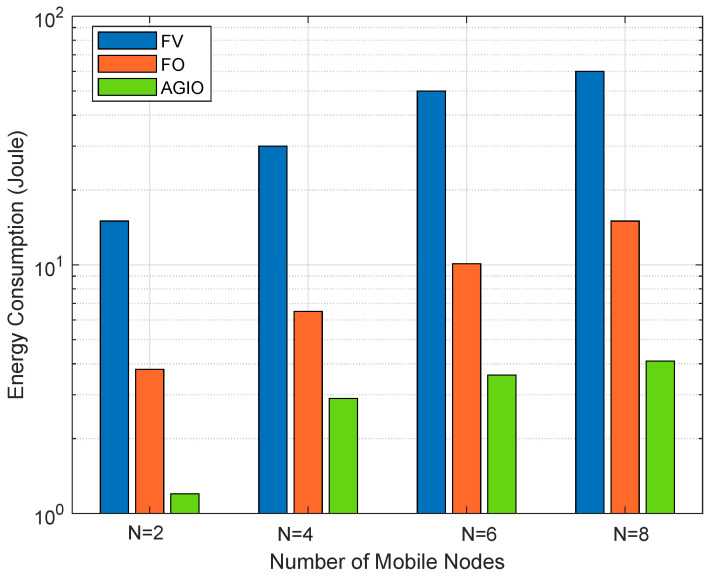
Comparison of energy consumption with a different number of mobile nodes.

Figure 4 compares the three different algorithms under different number of MNs. The energy consumption increases with N which is attributed to the increased number in computation tasks when the number of users increases. Again, the proposed AGIO algorithm outperforms both FV and FO algorithms. The superiority increases with the number of MNs, which reveals the applicability of the proposed algorithm in multi-user senarios. In addition, it is clear that the FO scheme outperforms the FV scheme, which mainly benefits from the optimal power allocation process in FO scheme.

**Figure 5:**
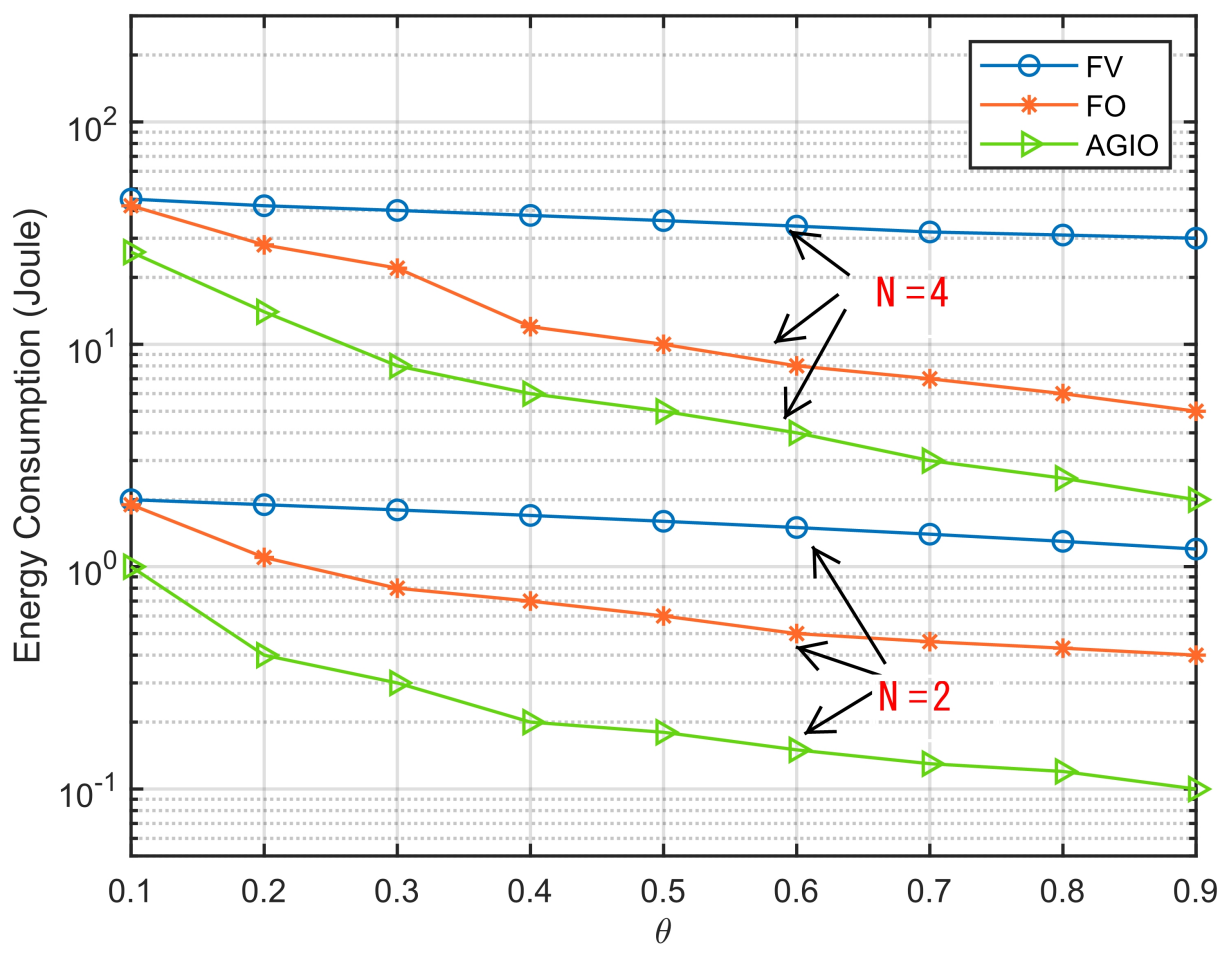
Comparison of energy consumption with different θ.

Figure 5 shows the energy consumption of the three algorithms under different harvest weight factor θ with N=2 and N=4, respectively. For the three algorithms, the energy consumption decreases with the parameter θ. The FO and AGIO algorithms demonstrate increased significant decline than FV due to the reason that θ represents the capability of energy harvesting and the greater value of θ means more harvested energy and less total system energy consumption. Thus, we can conclude MN tends to offload a larger proportion of computation bits to AN in the view of energy efficiency.

**Figure 6:**
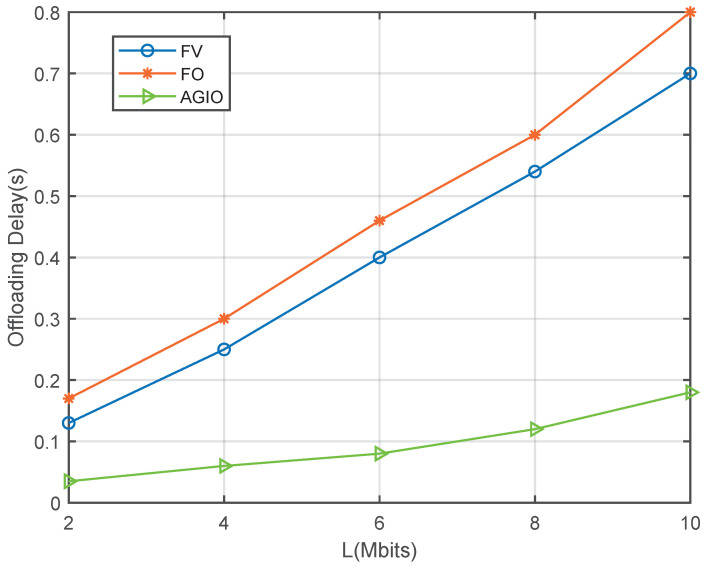
Comparison of offloading delay with different computation task size L.

Figure 6 demonstrates the offloading delay of MNs in the uplink transmission. The FV scheme shows a slight advantage than the FO algorithm because we set the offloading weight factor α=0.1 for FV, which means smaller offloading task in FV than in FO. Compared with the two benchmark algorithms, AGIO algorithm displays better latency performance due to the excellent design of optimization steps. It also indicates the distinguished feature of our algorithm both in energy consumption performance and latency performance.

### 5.2. Analysis and Discussion

Aiming at solving the data explosion and energy insufficiency challenges in WSN-assisted IoT system, we designed a novel framework that integrates MEC and SWIPT technologies into the IoT system. We formulate the energy consumption minimization problem and propose an AGIO algorithm to solve it. By jointly optimizing the CPU frequency, power allocation, offloading scheme and SWIPT scheme, we can achieve the minimum energy consumption.

Simulation results in Section 5.1 have verified our original intention. First, the proposed novel system shows great advantages in energy consumption and time delay, which are demonstrated in Figure 5 and Figure 6. It can attribute the success to more reasonable expressions of several parameters, which affects the system performance. That is also achieved by the first contribution listed in Section 1.3. In addition, the convergence result displayed in Figure 2 confirms the effectiveness of the AGIO algorithm as we stated in the second contribution. Furthermore, the simulation result in Figure 3 shows that the energy consumption decreases with the number of AN antennas. It provides a clue that our system is suitable for multi-antenna system, which is more efficient in applications. The simulation results in Figure 4 shows the superiority of the proposed algorithm in a multi-user scenario, which is exactly the practical application of a WSN-assisted IoT system.

## 6. Conclusions

In this paper, we investigate the wireless information transmission and energy transfer of a novel SWIPT-MEC enabled WSN-assisted IoT System. We fomulate an optimization problem by jointly optimizing the CPU frequency, transmitted power, offloading weight factor and harvest weight factor to achieve the minimum system energy consumption. In order to render the problem solvable, we propose a novel alternate group iteration optimization (AGIO) algorithm, which decomposes the original problem into three subproblems and alternately optimizes each subproblem using the group interior point iterative optimization algorithm. Finally, numerical simulation of the proposed strategy is carried on to compare with the two other benchmark schemes. The results demonstrate that the proposed design presents the performance advantages both in energy consumption and latency.

## Data Availability

The data used to support the findings of this study are available from the corresponding author upon request.
